# Undergraduate nursing students caring for cancer patients: hermeneutic phenomenological insights of their experiences

**DOI:** 10.1186/1472-6963-13-63

**Published:** 2013-02-15

**Authors:** Andreas Charalambous, Charis Kaite

**Affiliations:** 1 Head of the Euro-Mediterranean Research Centre for Oncology and Palliative Care, Cyprus University of Technology Department of Nursing, School of Health Sciences, Vragadinou 15, Limassol, 3041, Cyprus; 2 Department of Nursing, School of Health Sciences, Cyprus University of Technology, Vragadinou 15, Limassol, 3041, Cyprus

**Keywords:** Death, Nursing students, Cancer care, Reflective diaries, Clinical placements, Hermeneutic phenomenology

## Abstract

**Background:**

The care of patients suffering from cancer and especially those facing the death trajectory appears to be complex and demanding not only for student nurses but for professional nurses as well. The educational models often used in nursing require students to face challenging care scenarios, sometimes with minimal or no supervision and guidance. These “worst case scenarios” can be traumatic experiences that can leave the student hopeless and disappointed of themselves and in many cases can “scar” their subsequent professional career. The literature demonstrates that this can be the result of the students’ ill-preparation to care for cancer patients and deal with death and dying. The purpose of this study was to interpret the students’ experiences of coming face-to-face with cancer care during their clinical placements.

**Methods:**

This is a hermeneutic phenomenological study influenced by the ideas of the French Philosopher Paul Ricoeur. Based on this philosophical enquiry the interpretation process included three stages: 1) naïve reading, 2) structural analysis and 3) comprehensive understanding. Data were collected through reflective/narrative diaries from the 4^th^ grade undergraduate (pre-registration) nursing students practicing at oncology, hematology, pediatric oncology departments and hospices. Diaries of twelve students met the inclusion criteria and were included in the interpretation process. The study took place during January and May 2011.

**Results:**

The interpretation yielded the following themes: a) Being part of the center’s life, b) Being sympathetic, c) Being confronted by others, d) Being self-reflective, e) Being trapped in the system, f) Being caring towards the family and g) Being better in clinical practice.

**Conclusions:**

The students emphasized the need for appropriate preparation both at a theoretical and at a clinical level, as to better confront situations involving death and dying as well as learning techniques for crisis management. The students perceived the importance of adopting a policy that is both patient and family-centered in order to provide better care.

## Background

Cancer is a leading cause of death worldwide and accounted for 7.6 million deaths (13% of all deaths) in 2008 [1]. In Cyprus, the vast majority of cancer patients in 2007 aged 40+ had died from various forms of cancer [2]. According to the European Cancer Observatory, in Cyprus out of the 1154 men diagnosed with cancer, 148.7% had died from cancer whereas from 1059 women diagnosed with cancer, 90.5% had died from cancer [3].

Nurses are involved in caring for patients who are dying or have a terminal illness and are faced with the process of dying. Working with these patients and their families can be emotionally demanding and challenging. According to Birkholz et al. (2004) “death is a personal issue for each nurse and each nurse’s unique perspective can affect each patient who dies under the nurse’s care” (p.36) [4].

The International Council of Nurses [5] stresses that the nurses’ role is important when dealing with terminally ill patients in reducing suffering and improving the quality of life for patients and their families in the management of physical, social, psychological, spiritual and cultural needs. Nurses play an important role in developing a caring and supportive environment that acknowledges death in order to help family members to accept and deal with loss and grief [5].

The implementation of a hermeneutic phenomenological perspective allows the student nurses to describe their interactions with patients in the context which they occur allowing what is significant to the students to be revealed. Students are closely involved with patients and their families in different situations involving death and dying. Any learning in which they are engaged in is described in a narrative form that provides a visual image of their emotions, cognitions and perceptions [6]. Hermeneutics according to Ricoeur [7,8] is the theory of the workings of understanding in relation to text interpretation, because to interpret is to try to reveal description to itself that is to realize the meaning in human existence. Ricoeur [8] seeks for meanings behind the words and what is real is perceived in its totality.

There are studies on the nursing students’ experiences of caring for cancer patients revealing commonalities as well as differences regarding this topic. Whilst these studies are in their majority qualitative inquiries of the topic, they tend to retrieve data through interviews, questionnaires and even observations. It is the authors’ belief that data regarding the experiences of the students should also be collected through written narratives (diaries), a mean that allows the narrator to describe the experience in more detail without the pressure of an interview. Therefore, this study comes as a response to this gap in the literature.

A study conducted by Sadala and da Silva [9] in Brazil with fourteen undergraduate nursing students, aimed to understand how they perceive themselves while caring for terminal cancer patients and exposed the meaning of the experience they had. Findings showed that informants perceived it as a painful experience that made them confront their weaknesses and insecurities. They were feeling insecure and weak due to lack of preparation and inexperience as well as lack of support from professionals during their practical placement [9].

Cunningham et al. [10] conducted a study in London in order to explore student’s perceptions of their experience with cancer patients. They explored the sufficiency of their preparation for caring for cancer patients during their clinical placements. Data were collected through the use of a self-report questionnaire distributed to 152 pre-registration students enrolled on diploma degree nursing programme and follow up interviews with nine students. The majority of the informants (84.9%) described their experience as positive while the student’s perceptions of their confidence in practice (62.2%) were described as less positive. Similar data were reported in relation to their preparation for their required nursing skills (53.9%) as well as the amount of theory provided about cancer care before the clinical placements (38.2%). As far as their preparation of caring for cancer patients, student nurses claimed that they were feeling “being out of depth” meaning that they were not feeling comfortable as far as their education of communicating and supporting cancer patients. However, 93.3% of the informants were able to reflect on their nursing practice, 88% stated that they were feeling accepted as a team member whereas 81, 6% stated that they were being supported by their mentors in applying theory and practice [10].

Sanford et al. [11] in a recent study used a purposive sampling of 15 undergraduate Baccalaureate nursing students in three focus groups aiming to examine the experiences of nursing students caring for cancer patients in various cancer settings. Four sub-themes emerged in relation to student experiences: a) caring for patients and their families, b) interactions between students and healthcare providers, c) student experiences with dying patients, d) students prior experiences with cancer. Informants reported the lack of psychosocial care to patients by the staff nurses due to the limited time they had to spend with the patients. They also stressed the need for further training in order to face their own fears and prejudices and gain the necessary skills for being confident when providing care for cancer patients.

Huang et al. [12] in a descriptive qualitative study with 12 students in Taiwan explored the experiences of nursing students’ encountering death during their clinical practice. Findings showed that nursing students experienced feelings of terror and self-affirmation when providing care during the dying period as well as the difficulty of seeing patient’s suffering. Nursing students expressed the need for teaching and receiving support at the moment of a patient’s death and at the bereavement period that follows. Informants felt satisfied in relation to the knowledge and support gained from clinical mentors and nurses prior to patient’s death.

Allchin [13], in a hermeneutic study, identified and clarified the characteristics of students’ experiences in providing care for dying persons and their families. Data were collected from 12 nursing students who had cared for dying patients during the clinical rotation in adult health nursing. Three major themes emerged: a) initial hesitancy and discomfort, b) reflective nursing, and c) personal and professional benefits. Informants suggested the increase of both didactic and clinical end-of-life content in undergraduate nursing programs and the need for debriefing sessions for all students caring for the dying.

The aim of this study was to explore the experiences of the undergraduate students when they come face-to-face with cancer care during their clinical placements. The main objective being to help nursing students understand and interpret their experience.

## Method

### Design and setting

This is a hermeneutic phenomenological study following Ricoeur’s approach [14,15] in order to uncover the significance of experienced feelings and thoughts behind the students stories [16]. The main objective was not only to understand how nursing students’ experience their clinical placements but also to help them understand and interpret the situation of each patient. The texts are processed in order to capture the significance of the statements in the narratives [17]. The method of interpretation proceeded through three phases: a) naïve reading where the researcher does not perform any kind of analysis and thus acquires a first naïve guess of the meaning of the text within its context. The researcher reads the text several times and the surface understanding achieved provides direction for the next phase, the structural analysis. During the structural analysis, the connections and patterns of the text are uncovered by dividing the text into meaning units, condensed meaning units, themes and sub-themes [17]. These validate or invalidate the understanding gained from the naïve understanding. The final phase includes the comprehensive understanding, where the text is reviewed as a whole in light of the naïve reading and the structured analysis. The study took place during the academic year 2010–2011 and involved 4^th^ year nursing students practicing at oncology, hematology, pediatric oncology departments and hospices. The concept of “saturation” was the guiding principle on the decision in relation to the sample size. This term refers to the point at which no new information, themes or sub-themes are observed in the data [18].

### Informants

Students’ participation was based on their willingness to take part in the study. The students with prior experience in oncology, hematology, pediatric oncology departments and hospices were excluded. The reason lays in the aim of the study to explore the first encounters of the students with care taking place in such clinical settings. Once, informants were selected, they were informed in detail about the current study and a written consent was retrieved.

### Data collection

The reflective diaries were used to elicit the research data with a narrative approach (unstructured recordings) that emphasized on the description of the experience through the use of “texts”. Ricoeur [7] stressed that text displays a fundamental characteristic of the historicity of human experience, namely that it is communication in and through distance. In this study, the hermeneutic (interpretive) task was to elicit the textual-basis for the interpretation of the students’ experiences. This task becomes legitimate and feasible through the use of reflective diaries since these are able to extract “text” to which apparent meaning can be attributed to. The hermeneutical task for Ricoeur [8] is “the work of thought which consists in deciphering the hidden meaning in the apparent meaning, in unfolding the levels of meaning implied in the literal meaning”. Here hermeneutics makes contact with its older exegetical tradition in the task of deciphering texts.

This being a hermeneutic phenomenological study inspired by the philosophical ideas of Paul Ricoeur the “text”, whether written or verbal, is the primary focus of the interpretation and the portal that opens up the understanding of the experience. On this topic, Ricoeur argues in his *Interpretation Theory: Discourse and the Surplus of Meaning*[8] that it is “only writing in freeing itself, not only from its author and from its originary audience, but from the narrowness of the dialogical situation, reveals this destination of discourse as projecting a world” (p.37). However, at this point Ricoeur resisted the temptation to reduce hermeneutics to a purely linguistic analysis of meaning or the “ideology of an absolute text” as he calls it. Kearney [19] asserts that Ricoeur does not wish to deny that as soon as discourse is inscribed in a text, the author’s intention ceases to coincide with the meaning of this text. “What the text says now matters more than what the author meant to say and every exegesis unfolds its procedures within the circumference of a meaning that has broken its mooring to the psychology of the author” [36, p24].

Therefore, the decision to implement a written narrative approach as the means to explore the students’ experiences lies on the fact that this approach is a text-orientated interpretation, and inasmuch as text are, among other things, instances of written language. What happens in writing according to Ricoeur [8] is the “full manifestation of something that is in a virtual state, something nascent and inchoate, in living speech, namely the detachment of meaning from the event…writing is the full manifestation of discourse” (p.25).

Previously to participating in the study, the students received a 2-h session introducing the use of reflective diaries followed by several practical exercises with the use of various clinical scenarios. The exercises were repeated until the students have become competent at this task. As part of this study, students were asked to record in writing insights with regards to the following themes: feelings, perceptions, concerns, fears and experiences of their first encounters with the clinical settings and the care itself. Although, these themes were the focus of the diaries, no specific questions were used to guide the students as to their testimonies. Furthermore, in order to minimize the influence of the authors on the collected textual descriptions, the students were given the opportunity to record their experiences in a freely and unstructured way (narration), allowing them to elucidate them in more depth. This unstructured strategy gave the opportunity to seemingly unimportant issues (to those experiencing an event as outsiders and to the reader) and unvoiced experiences to surface in the students’ writings.

The students recorded their experiences during their clinical practice, twice a week and within a period of 6 weeks. The immediate recordings of the events facilitated the detailed description of the experience without allowing the gaps in the memory negatively influence the production of the text. One of the reasons for choosing to use the diaries was the opportunity provided to students to recall the event at an ease of time, which would allow a more accurate passage of the experience on text. No word limit was imposed on the informants as to their records and they were encouraged to emphasize on the constituent elements of the actual experience. These in-depth details of their descriptions facilitated the deconstruction of the patients’ experience and opened up the meaning to a wider audience (namely the readers) and context. Only the reflective diaries that included consisting recordings of the students experiences were considered for analysis. In total 12 reflective diaries met the inclusion criteria.

### Data interpretation, rigor and methodological reflections

This being a hermeneutic phenomenological study of the experiences of students encountering the nature of caring for cancer patients, an interpretive process was considered appropriate. This method has been used in several preceding studies with a nursing perspective [6,13,15,18].

Dempsey and Dempsey [20] state that the terms validity, reliability and generalisability are generally avoided in qualitative research and qualitative researchers prefer to use the terms “truth and accuracy”. One critical question in qualitative inquiry concerns the knowledge claim that can be made. Are the findings limited to the informants included in the study, or can the findings be generalised or transferred or are they applicable to other contexts or other people? The results of this study can not be generalised if one employs a statistical definition of generalisability dependent on sample size and representativity. However, if generalisability is understood as being on a theoretical and conceptual level, the understanding gained from this study may be transferable to people in contexts beyond those that were studied. Hopefully, the findings can convey understandings and descriptions enabling readers to see their own situation in a new and comprehensive way.

Another critical question is on what grounds we can believe the findings of a qualitative study. Different methodological traditions have somewhat different arguments for the credibility of qualitative findings. When it comes to hermeneutic phenomenology, Ricoeur [8] asserts that it is always possible to argue either for or against an interpretation, and the credibility of an interpretation lies closer to the logic of probability than to the logic of empirical verification. Credibility is argumentative and is established in a discourse. The findings of a study achieve credibility when other researchers come to regard them as sufficiently trustworthy to rely on in their own work [21]. Bruner [22] asserts that credibility in narrative research can also be understood as verisimilitude, meaning that the interpretations appear truthful in comparison to one’s own experiences and in relation to shared meanings in society.

### Ethical considerations

The students were guaranteed anonymity in the reporting of the study’s findings. All the reflective diaries were kept safe in a locker at the Cyprus University of Technology with restricted access. The diaries were kept separately from the informants’ demographic data to ensure anonymity. Any potential participant retained his/her right to withdraw from the study without any repercussions. Appropriate ethical approval was obtained.

## Results

### Interpretive perspectives

The findings presented here are the end product of a circular movement between the three phases of interpretation namely the naïve reading, the structural analysis and the comprehensive understanding. For the interpretation to be complete, the principle of the *hermeneutic arc*, as this was proposed by Ricoeur [14] was intergraded in the interpretation process. Ricoeur [14] used this term to describe the movement back and forth between a naïve and an in-depth interpretation.

#### Naïve reading

A first conjecture, i.e. naïve understanding, was formulated of providing care to patients suffering from cancer which appears to be an unpleasant experience for the undergraduate students. Students narrated discomforting and demanding situations where they found themselves “trapped” leaving them exposed to the patients and their families. They acknowledge that their inability to respond to these scenarios was complex with many contributing factors such as the ill-education and preparation, the context in which care is delivered and the insufficient or lack of clinical guidance by professional nurses.

#### Structural analyses

The structural analyses are directed towards the structure of the text, and towards establishing whether the structure invalidates or validates the preliminary interpretations of the whole as these were manifested in the naïve reading phase. The structural analyses produced the following six themes and each theme is explored through a number of sub-themes and main points are illustrated by anonymous quotations: a) Being part of the center’s life, b) Being sympathetic, c) Being confronted by others, d) Being self-reflective, e) Being trapped in the system, f) Being caring towards the family and g) Being better in clinical practice.

### Being part of the center’s life

The student informants reported a collaborative environment in some centers, providing holistic care as well as supporting nursing staff. Informants also reflected the feeling of a “family”, meaning that patients and staff are conceived as a family. This interpersonal relationship developed between the nurse and the patient is considered by the students as a “role model” practice based on which their caring should be based on. The students as observers learn from this practice which assists them in becoming better nurses.

#### Sub-theme: being part of the “family”

**M2:** “Here […] the person is not merely a “number”…everybody is known by their names… Is our Marios, our Chrysanthi, and our Christos. Our Elena, the mother of Yiannis and Mrs. Maria the grandmother of our Michalis. Most members of the staff appear so connected with the patients’ and the families’, they all seem to deal with the (life-threatening) situation as one… It certainly feels so different than the wards I have worked so far…it is as if the “patient” is the family as a whole… It′s the person in his entirety, with the personality, his illness and with anything else that defines him. You need to give importance to all…every little detail matters…You do not omit anything”. This experience has been life changing for me as it made it obvious of how I should behave in my practice, certainly this is and will be the model for my professional career, I just like to become a “member’ of this family”.

**M1**: “No more lies, no more theory…it all comes to this…everyone want to be treated as a member of a family, this has made me realize an important aspect today…becoming a better nurse means seeing and treating the human being not the disease, this is what it takes”.

### Being sympathetic

Nursing students expressed mixed feelings towards patients. Students commented on feelings of sorrow and disappointment due to the patients’ situation and the fact that they suffered from a terminal illness. An observation that stood out in the narrative was the comprehension and experience of the stages of grief that patients are going through. Nursing students often felt puzzled as to how they could help patients cope with the stages of grief.

#### Sub-theme: “being shattered in pieces”

**B1**: “Frankly, in certain moments I felt disappointed and shattered witnessing various situations, where the patients were daily confronted with high levels of depression and stress… on one hand the patients have to deal with their own health problems and on the other hand they had to deal with their families, who in most cases were in denial… I have observed that proportional problems were brought forwarded by the persons of their environment, such as relatives or friends who had assumed their care”.

#### Sub-theme: being an “emotional roller coaster”

**G7: “**Feelings of affection, support, sincere humility, love and support overwhelmed me each time I am working on this ward. Every time there is a different person, each one in a different stage of the confrontation of death. I simply experience, what they experience, to an extent at least…with the “ups” and “downs”, the good days and the bad days, the days I am optimistic and the days I am pessimistic”.

**M8:** “I spent the morning not with a patient, but with the wife of a deceased patient (died last week). She wanted to talk to someone and I was the last person in the room with her husband just before he past away…I felt connected with her and the rest of the family during those last hours…it is difficult to describe the “how” or the “why”…it is just that happened…so we spent the morning together…I listen mostly to her…I just wish I could help her more”.

### Being confronted by others

Students during their participative observation in the wards experienced two distinctive behavioral pathways that challenged their beliefs and their theoretical education. These included nurses who behaved in an offensive and less caring way towards the patients and simultaneously deprived the students from acting upon such situations. The students experienced this behaviour as an underestimation of their abilities to interact with the patients and an exercise of power over them by the professional nurses. Students felt powerless in their practice and constrained in their duties. However, the students also identified nursing behaviors that included the necessary support to patients and their families. This group of nurses also acted as role models for the students, adopting this way a more responsible and mentorship-like approach.

#### Sub-theme: being challenged and powerless

**M1:** “We observed many times that the staff had behaved offensively towards patients, without respecting the unfavorable position in which they were. Unfortunately, many times, we, as students seek to approach the patient, but sometimes, the personnel looks at us curiously because they consider that communication with the patient is a loss of time. They simply do not have faith in our abilities to approach and effectively communicate with the patient in suffering, it is so disappointing”.

**F6:** “It is not only that they do not have faith in us…I get the impression that they rather are “afraid” of us, I don’t mean it in a real sense, but as intruders we invade their personal space their *kingdom* where they rule and feel challenged… they feel that they need to be protected against us and the easiest way to achieve this is by exercising their power over us…and this can take many forms”.

**F5**: “It is a dull and “cold” center; it almost gives me the creeps. The confrontation that we had from the staff was disappointing. Some of them made us feel as if we were a burden, an unnecessary but mandatory issue that they had to deal with during their work. They were moments that I felt so bad; I preferred to be anywhere else but here […]. Not trusting us meant that they did not trust our abilities and skills…they somewhat took for granted that we had nothing to offer”.

#### Sub-theme: being a self-actualized nurse

**G7:** “With her (i.e. professional nurse), I have learned much, the way one should work and treat each patient as a unique individual […] paying attention to (important) details. Everything is important and relevant to caring, nothing should be assumed. The nurse X was caring for the patients with love and affection. I would have wanted to be one nurse just like her, worthy, with self confidence, creative and very energetic. This is a nurse every patient wants be cared by”.

**B1**: “I was training in this ward for 2 days and I felt that what I had been taught in the classroom was in the best case not applicable in practice. My whole training was challenged and I found myself in uncomforting situations. Many times I had to rely on myself to cope with the situation; I had to drawn on good examples from my practice and avoid the bad ones”.

### Being self-reflective

During their clinical practical nursing students evaluated their own abilities, behaviors and reactions towards the patients. Feelings of self fulfillment when accomplishing a task were reported by the majority of the student informants and this was generally observed when good communication with the patients was established. Not paying attention to details communicated by the patients made patients and nursing students feeling uncomfortable. The same problem occurred when nursing students were not in a position to accept their own fears and prejudices especially in relation to death and dying.

#### Sub-theme: being acknowledged

**Β2: “**Ηis mother told me: you have moved us, you are suitable to be a nurse. You will become a very good nurse.” Before I left, his father had shaken my hand and told me thank you for everything…it made me feel so good about my work, about the “good” I was offering to the patient…now I am sure that I made the right choice, I am in the right place to actually make a difference”.

**B3:** “I am not celebrating merely because I have done something good, it is simply my job, what I was trained to do best, I don’t expect congratulations from everybody, just acknowledgement for what I do…and the patients and their families are generous with that, I don’t know if I always deserve it…but it sure makes a difference when it comes”.

**G6:** “They [the patients] kept thanking us for insignificant things… That was particularly impressing for me… They showed particular interest about us. They wanted to know about our lives, where we came from, about our studies and many more. They demonstrated a genuine interest, and this was rewarding. They kept wishing us good follow up with our studies and be good practitioners because they relied on us to care for them”.

#### Sub-theme: being lost in translation

**G4**: “Today, while we were taking the medical history from a female patient, I wasn’t careful that she had a mutilated right leg and asked her if she could easily move. She said: *How?* I felt so bad and embarrassed; I wanted to vanish from the face of earth…why I wasn’t more careful? I learned the hard way that is important to be a good observer and listener and that our patients expect us to be as such”.

**M1**: “He was trying to say something to me…I didn’t figure it out until now…he didn’t feel comfortable having his family around when the doctor gave the news…I didn’t realize…I took some thing for granted and that was obviously wrong!”.

#### Sub-theme: being frightened and challenged by cancer

**G6:** […] “I knew that a patient would die and I was trembling on the idea that this might occur on my presence in the room, I felt fear and at the same time sorrow just by thinking of it. When leaving, I thought that I had to accept that death is part of our lives, part of our job and that those people had the need to be listened to and be supported by me. I believe that what made me feel horrified was what I would do if one of my loved ones was in a similar situation or what I would have done if I was dying of cancer…?. These thoughts circled my mind for weeks…At the end of the day I was trying to be in their shoes and this was draining away all of my strengths”.

### Being “trapped” in the system

Having a different confrontation on patient’s situations was reported by the student nurses. Nurses’ burnout and not showing empathy towards the patients were among the important issues that students acknowledged during their clinical practice. They also stressed that there was a strong presence of prejudice that driven the actions of some people working in the hospital. Students acknowledged that prejudice also influenced their own practices.

#### Sub-theme: being driven by prejudice

**G5:** “Often in practice, we take for granted many things, especially health related information. However, knowledge and information seem that are not so well distributed among the staff working in hospitals. I remember, one child with herpes zoster kept ringing the bell for taking his milk and no one was responding. I was impressed when I entered the room facing the dishes that he ate the day before. This means that cleaners were not well informed by us or the nurses and the fear of transmission kept them from entering the room. It is neither acceptable nor fair to the patient having to pay the results of ill informing processes of the staff or the prejudices some people hold against certain illnesses”.

**B1:** “Having cancer is not a simple thing, it has never been in Cyprus, and we still come across and have to deal with examples of prejudice against cancer itself. The taboo remains strong even among us students sometimes but we have a duty to set things right, at least to the extent that our knowledge and power allows us to do”.

#### Sub-theme: being emotionally detached

**G1:** “During the nursing round, we came across a patient that was in a critical state. That moment a nurse that cared for him the day before said: *He has not died yet? I thought that I will come today morning and I wouldn’t find him alive […]* That moment I thought of replying to her…but I restrained myself from doing so […] I was so mad with myself for not saying nothing, after all she (i.e. the nurse) overruled everything that I have learned in theory and gave a bad impression of what she represents…the nursing itself. I am still wondering how a person can be so crude or insensitive to another person.”

**M1**: ‘Listen [a male nurse talking to a group of students]…this is not an easy ward to work in…it takes courage and strength to be able to handle the physical and emotional burden of caring…you cannot feel sorry and bereaved for every patient you care for…you have to be in a ‘safe’ distance…otherwise it is you that will eventually become the patient. At first I thought that this sounded perfectly logic, however, I soon had to reject this approach because I had realized that nursing is all about human contact and human interaction, what’s left if you take away these prominent principles?.’

### Being caring towards the family

Cancer is a blow to every family and each person/significant other reacts to differently. Many feelings that are faced by patients are also faced by relatives. The stages of grief are a process that relatives go through in order to cope after the diagnosis and includes the following stages: a) denial, b) anger, c) bargaining, d) depression and e) acceptance. Everyone experiences them in a different way and sometime in a different order, than the one suggested by Kübler Ross [23]. Reactions to illness, death and loss are as unique as the person experiencing them. Some of the relatives are trying to be cheerful in front of the patient acting as normal in order not to influence patient’s psychology and empower them. However, when being on their one their true emotions emerge, often leaving those crying and stranded in a prison like situation, relying only on themselves to cope. Anger is the second stage of the stages of grief. The individual recognizes that denial cannot continue and because of anger, the person is very difficult to care for due to misplaced feelings of rage and envy. There were relatives reporting that health professionals are not giving the appropriate attention to the psychological aspect of the disease and support alternative therapies. There were relatives reporting changes in their energy level, as signs of depression.

#### Sub-theme: being emotionally and psychologically supportive

B3: “Parents expressed certain complaints for health professionals in Cyprus that they tend to focus on the biological aspects of their children, while the psychological aspects are left untouched and often neglected. Even if health professionals are paying attention to it, this is to a minimal degree. Those parents were supporters of alternative treatments and medicines”.

#### Sub-theme: being let down

B5: “During the day I was upset with the words of a father that he stated that he was tired. At first, I felt disappointed but later on, I figured out that maybe due to the stress and worries for his child’s health he had reacted in this manner. Instead of being judgmental, I should have realized that this father and every father in his shoes is in need for my support now more than ever, being there when mostly needed […]”.

#### Sub-theme: βeing offensive towards the nurses

G4: “I went to take the vital signs of a lady that she was dying and she did not want to, I have explained to her why this was necessary and she finally agreed to it. A relative of her then told me in a very offensive manner: *She is afraid of you and she does not accept anything from you*. Holy Mary, I felt so bad, and of course I did not response to his remarks. . What does it mean that she was afraid of us? Because we may be doing something painful for her own good this does not mean that we care? But from those people that they say *when she dies call us to take her* what do you expect? I do not want to get in touch with those people and of course I don’t want them to have such an impression for me, and nurses in general”.

### Being better for clinical practice

Student informants in their narratives have identified issues that they considered as problematic in clinical practice. With their objective observational role, they frequently took the opportunity to challenge some of the circumstances under which care was offered to cancer patients and made recommendations towards improving the overall experience of the patient.

#### Sub-theme: being a holistic carer

**B1: “**I do not know, but generally I believe that while a successful achievement in fighting cancer has been achieved for the bodily aspect of the problem, on the contrary for the psychological and emotional support there are a lot of serious gaps. Communication with the patient and his psychological support are issues that definitely need more attention. The administration of the hospital must play a substantial role in this objective by practicing an effective policy which will be focused on each patient as a unique entity with individualized needs and concerns with a focus on his/her physiological and social needs”.

#### Sub-theme: changing the color of the nursing uniform

**B3: “**“I think hat it would be good for pediatric wards not to have white uniforms for their nursing staff but instead more colorful uniforms. This lays on the fact that the white gown tends to trigger fear among the children. Whether we like it or not the children have matched the white uniform with uncomfortable and often painful procedures, a more colorful uniform will most likely create a better mood for the children in suffering”.

#### Sub-theme: being creative

**G5:** “I believe that in this ward, more activities should have been offered especially for teenage patients because these can act as means to distract their thoughts of their health problems. Unfortunately, at the moment, the ward offers no alternative activities. It feels as if we have let the patients do everything by themselves, without support. I think that this is something that needs to be changed”.

### Comprehensive understanding

During this phase, the interpretation cycle is completed with the critical reflections on the researchers’ pre-understandings, the sense of the naive reading, the findings from the structural analyses, the research question, the context of the study, and the relevant literature in order to gain a deeper understanding. This deeper understanding is the decipher meaning of what the students narrated in their reflective diaries in relation to being caring for a patient suffering from cancer.

The theme “Being part of the center’s life” according to the interpretation has been related with the way informants reflected the feelings generated by their presence in the various clinical centers. Even though the informants considered themselves as being part of the organization, they often felt neglected and in a way “punished”. However, this did not negatively influence the development of strong relationships with the patients and their families. On this issue, the informants repeatedly expressed their concern on the ineffective or ill communication between the health care professionals and the patient and the family. The issue of communication was reported in the study of Sanford et al. [11] where the lack of communication between the patient and the healthcare professionals was conceived as lack of caring. This interpretation becomes more probable if we take into consideration the fact that the patients rely on the healthcare professionals and especially the nurses for information on their situation.

The next theme entitled “being sympathetic”, encapsulated the informants’ ambivalent feelings towards patients as well as feeling puzzled as to how helping the patients cope with the stages of grief. The inability of the nursing students to cope with the care of terminally ill patients lies on their inadequate clinical and theoretical preparation and is in line with the findings of the study of Cunningham et al. [10]. According to the researchers, the informants reported less positive feelings about their confidence in practice and their preparation for the required nursing skills as well as the amount of theory provided about cancer care before their clinical placements. Further findings are in line with the study of Sanford et al. [11] as well as the study of Allchin [13] and Sadala and da Silva [9] where nursing students were feeling a tension between what they believed that should be done to help the patient and what they were able to do due to lack of preparation and inability to cope with their own feelings. Reflecting on the Cypriot clinical setting, the students are often found trapped in situations that can generate feelings of disappointment, distress, despair and anger due to the inability or the ill-preparedness of the student to handle such situations. This is perhaps the result of the lack or poor mentorship for 4^th^ year students in the clinical oncology settings, as these students are considered able to work with the patient with minimal supervision or guidance.

“Being confronted by others” was conceived both as positive and as negative aspect in some of the centers. Students identified members of the staff that underestimated their clinical skills and further considered them as less supportive during their clinical practice. They assert that they often provided poor guidance to the students which let the student to unpleasant clinical experiences involving the inability to effectively respond to the patient’s or the family’s needs. Contrary, the staff that was identified as supportive by the informants, acted as role model and a key player for students’ learning whilst in clinical practice. They were perceived as “open”, “approachable” and “understanding” towards the students’ needs and concerns. The positive effect of supportive professionals was emphasized in the study of Cunningham et al. [10] where most of the nursing students reported the importance of support during their clinical practice. This aspect facilitated their clinical experiences and promoted their abilities to provide efficient care to the patients tackling difficult situations. On the contrary, in the studies of Huang et al. [12] and http://Saarikoski et al. [24], nursing students reported that during the period of caring for near-death patients, mentors and nurses were providing guidance and support in terms of preparing the students caring for terminally ill patients while little help was given during the dying process and the bereavement phase. The lack of support resulted in struggling to manage their feelings and ill-prepared them to offer support for the loved ones left behind.

Reflecting on their abilities and responses towards patients revealed that in their majority students expressed feelings of self-fulfillment when accomplishing a task. Informants emphasized the importance of good communication with the patients in clinical practice and acknowledged that managing to establish good communication could reflect their self actualization. Within the study of Sanford et al. [11] students commented that the interactions with the patients were rewarding to them. On the contrary, the poor or lack of communication was reported as a complication in the care of the patients. Cunningham et al. [10] in their study showed that lack of communication from the informants was expressed in the form of fear and inadequacies, a manifestation also recorder in this study. Similar finding are reported in the study of Sadala and da Silva [9] where the nursing students’ inexperience of dealing with stressful situations such as the care of terminally ill patients was conceived as a communication barrier that blocked the effective delivery of the care.

The issue of providing good or ill communication also prominent in the theme related to the student nurses’ evaluation of their interventions to the patients. The provision of inadequate communication and the inability of nursing students to deal with their own perceptions of cancer and thoughts of death were outlined in the study of Sanford et al. [11].

The reported emotional exhaustion of the students that emerged as a finding of the current study stresses the necessity of high-quality preparation and support to both professional nurses and nursing students in relation to the care of terminally ill patients [9,12,13]. The emotional depletion seemed to be experienced by the students as a situation that they could not escape from. This assumption was based on the described occasions where the challenging experiences of the students influenced their family and social interactions. Caring deeply, as these students did, about a person who suffers from cancer was experienced as a limit situation in life, sometimes an unbearable situation that was difficult to be resolved. This findings emphasizes the role of the broad meaning of providing effective or ineffective cancer care which can also be related to patient satisfaction as well as to nursing student’s satisfaction of being able to implement in clinical practice their knowledge and skills [9].

“Being frightened and challenged by cancer” is a hidden aspect of being a student nurse or professional nurse working in Cyprus. The aspect that seems to generate the fear is a possible confrontation with a patient suffering from cancer that in a way relates to them through family or social connections. Assuming the role of the caregiver in these situations can be burdensome and often can lead to excess stress, disempowerment and lack of effectiveness in their role [25,26]. The cultural aspect of cancer as a taboo should also be considered in these situations since largely cancer is connected to death and dying [18]. Being diagnosed with cancer brings up many emotions both for the patient and his/her family. The possibility of dying brings up more intense and overwhelming feelings. Student nurses reported that cancer for some patients was synonymous to death and this was expressed in everyday life by the majority of the patients. However, other patients chose to suffer in silence, but their depression and sadness was graphically expressed on their faces. Fear of dying, distress and insecurity after being diagnosed with cancer or living with cancer are topics often students and nurses are called to act upon. Many patients cannot even pronounce the word cancer due to their intense worries of death and dying. Some fears are solely based on rumors, outdated information and sociocultural norms. In Cyprus few people can say that they or a member of their family has been diagnosed with cancer or has died from cancer. The fear of not being able to take care of their own family often drives the patients to deny informing their relatives about their disease in order to ‘protect’ them from the possibility of setting aside their own lives to care for the patient. Sadness and depression are also reported as a normal response to any life-threatening disease including cancer. However despite the obvious stereotypes, students and nurses can act as catalysts to facilitate the patients and their families to better deal with the changes and the new realitied imposed by a life-threatening disease. For many of the students, cancer is considered a terminally ill disease and therefore death and cancer have somewhat became synonymous with many negative cannotations deriving as a result. This generates the feelings and fear of death not only among students but also among professional nurses [18]. As cancer is considered a taboo topic openly communicating about cancer is often avoided [18]. The patients belonging to the same cultural community demonstrated a similar response. Therefore, in the journals the students narrated situations where they exorcized the disease with expressions such as the “damned disease” (*katarameni astheneia*) or the “evil” or the “that thing”. This being a negative prejudice towards the disease itself, it can also be transferred toward the patient and its’ care strengthening this way their inability to cope and promoting “avoidance” as a defensive mechanism especially in those cases where the patient is a person socially close to them.

Being caring for the family was a theme identified in the current study that was strongly related with the stages of grief that relatives go through, in order to cope after the diagnosis that includes the stages suggested and identified by Kubler Ross [23]. The theme emphasized the fact that cancer remains a “social disease” that does not affect only the patient but the family as a whole. This stresses the need for a holistic approach involving the family in the care and not just the patient in all aspects of the disease trajectory. The fact that health professionals were giving inadequate attention to the emotional and social problems generated by cancer was a fact stressed in the study of Sanford et al. [11]. The researchers found that the nursing students reported that due to limitation of time and the need for further training of health professionals little attention was given to the psychosocial care of the patients and their families. This finding links to health professional’s reported low self esteem which is attributed to the lack of skills and knowledge when providing care for cancer patients as well as their prejudice towards cancer which can be manifested as avoidance [18].

Within the theme “Being better for clinical practice” the student informants stressed the need for emotional and psychological support for both the patient and the family. They also commented on the need for the adoption of a policy that focuses on the patient as the center of the care. The students have repeatedly acknowledged in their daily reflections, the importance of patient-centered care in clinical practice. As a prominent example of this need, the students commented on the need for patient-centered communication between the healthcare professional and the patient. The topic of patient-centered care has received extensive attention in the literature not only by the students’ perspective [27], but also by the perspective of the professional nurse [28,29] and the clinicians [30,31]. In relation to the suggestion made by the student informants, these were focused in relation to the nurses’ uniforms and the provision of more creative activities to teenage patients. The study by Meyer [32] explored children’s’ perceptions of nurse caregivers based on uniform color and style. The children that participated in this study preferred the nurse wearing a colorful smock top, and most feared the nurse wearing a white dress uniform. This conclusion coincides with some of the expressed perspectives of the students that argued in favor of a more colorful uniform instead of a white one. Similar studies have explored this issue in adult patient with contradicting however results [33,34].

## Discussion

The ill preparation of students has been identified in the current study and points out the need for more theoretical and experiential laboratories as well as the formation of debriefing sessions after the students’ clinical placement. Additionally to better education, researchers need to focus on those aspects identified by this study that have the potential to increase the willingness of the student nurses to care for cancer patients and their families. Preceding studies have also identified these approaches as possible means to improve the clinical experiences of student nurses in cancer care settings [10,13,35]. The promotion of crisis management for nursing students is important prior to the clinical placement through appropriate education such as role playing. The use of educational scenarios (i.e. death and dying scenarios, angry relative, bereaved relative) on which students can reflect on can facilitate their better preparation for interacting with patients and their families by developing skills for providing comfort, responding to anger, responding to other nursing staff as well as responding to the family’s needs. During the clinical placement it is important to have a mentor that would support the nursing students in these cases and help the students overcome any painful experiences when facing for example a patient’s death [12]. The education of nursing staff in mentoring nursing students as well as supporting them in such situations is also recommended in line with the studies of Cunningham et al. [10] and Sadala and da Silva [9]. Another, aspect that was highlighted by this study was the negative impact of some of the professional nurses on how cancer care was practiced. However, a strategy that could be used to challenge the negative attitudes of the professional could be their active involvement in the planning of the students’ clinical education and training as advocators. By providing them with the opportunity to voice their views and concerns on how students should be trained could positively influence their own practice and potentially improve their interactions with the students in the future. In this shared environment studies such as this one can be used as an important feedback tool for both professional and student nurses for improving clinical practices.

Hermeneutic phenomenology was applied as the methodological and conceptual framework for this study. Ricoeur’s philosophical approach was applied in all the stages of conducting the research and interpreting the results. The method allowed the researcher to understand the nature of the experience, making visible own pre-conceptions and pre-understanding of the phenomenon. Reflective diaries gave the opportunity to the researchers to critique the insight of the transcripts, bringing at the same time to the interpretation the researchers own experiences.

The review of the relevant literature demonstrated that findings are in line with the findings of earlier studies that lack of preparation before the clinical placement in cancer settings is a source of feelings of insecurity and fear that provoke the good communication of nursing students and patients as well as their significant others which further acts as a blocker to the provision of quality of care to cancer patients. Lack of information creates prejudices that link to social misrepresentations of cancer which lead to exaggerated responses to the disease and makes it more difficult both for the healthcare professional to cope as well as for the relatives to step to the stage of acceptance.

This is an innovative study for Cyprus as it gives for the first time the insights of nursing student’s reflective diaries and is the first step for conducting further research on the subject matter in order to improve the nursing education on cancer care in Cyprus as well as deal with prejudices for the benefit of both the patient and the family.

### Limitations

One single study cannot investigate the phenomenon to its full extent. The themes and sub-themes highlighted in this study offer opportunities for further research in the field covering a broad area of concerns. Exploring the experience of nursing staff acting as mentors for the students could bring a deeper inside to the topic. Furthermore, a research study using anonymously those reflective journals for group discussion could enhance the nursing student’s preparation for future work in cancer care settings. The use of focus groups and the triangulation of data through individual interviews or narratives as well as the observation of nursing student’s clinical placement in combination to reflective diaries could also give a broader insight of the experiences of nursing staff, nursing students as well as patients and their families. The reflective diaries are able to elicit written testimonies on the experiences of people, since for Ricoeur [36] “text is discourse fixed in writing” (p145), and have an apparent advantage over interviews. Text produced through reflective diaries can be interpreted without further processing by the interpreter, whilst narratives (discourse), can not produce meaning until they are transformed into text. Ricoeur [36] endeavored to make clear which traits of discourse can be altered by the passage from speech to writing. He argued that discourse, being an event occurring at a particular point of time, is not preserved entirely unchanged when committed to written form such as interview transcripts. However, the combination of methods could allow for some of the weaknesses in collecting the data, to be addressed. The main aspect that needs consideration in future studies is the “phenomenological” understanding of the experiences interpreted in this study through the sole use of diaries, an aspect that could possibly be better addressed through the combination of extensive written and verbal transcripts.

In any hermeneutic phenomenological study of a phenomenon, there is perhaps a question that repeatedly comes up, that of the generalisability of the findings and their applicability to similar contexts [37]. This has been discussed earlier in the paper, however a notion introduced and discussed by Ricoeur also needs to be acknowledged here. This is the notion of “multiplicity” of language, the fact that the experiences can be manifested into different interpretations. These are the different ways by which the text opens up to the reader/interpreter, and therefore not all interpretations are equally truth or probable. With this point of view he acknowledges the polysemy of language and recognizes the legitimacy of many different meanings and conflicting views.

## Conclusion

The importance of understanding the experiences of nursing students helped both the nursing students to have an insight and meaningful interpretation of their clinical placement in order to be prepared for future work in the oncology wards, give feedback and recommendation for clinical practice, nursing education, nursing policy as well as patients and significant others for confronting issues of death and dying. These findings can serve as a mean to change the focus of the theoretical preparation of nursing students as to facilitate their clinical practice experience.

## Competing interests

The authors have no competing interests to declare.

## Authors’ contributions

The main idea of this study belongs to CA. All authors (CA and CK) participated in all parts of the preparation and submission of the manuscript. All authors (CA and CK) read and approved the final manuscript.

## Pre-publication history

The pre-publication history for this paper can be accessed here:

http://www.biomedcentral.com/1472-6963/13/63/prepub
